# Moving toward a contemporary classification of drug-induced kidney disease

**DOI:** 10.1186/s13054-023-04720-2

**Published:** 2023-11-09

**Authors:** Iman Karimzadeh, Erin F. Barreto, John A. Kellum, Linda Awdishu, Patrick T. Murray, Marlies Ostermann, Azra Bihorac, Ravindra L. Mehta, Stuart L. Goldstein, Kianoush B. Kashani, Sandra L. Kane-Gill

**Affiliations:** 1 Department of Clinical Pharmacy, School of Pharmacy, Shiraz University of Medical Sciences, Shiraz, Iran; 2 Department of Pharmacy, Mayo Clinic, Rochester, MN USA; 3 Department of Critical Care Medicine, Center for Critical Care Nephrology, University of Pittsburgh School of Medicine, Pittsburgh, PA USA; 4 Division of Clinical Pharmacy, San Diego Skaggs School of Pharmacy and Pharmaceutical Sciences, University of California, La Jolla, CA USA; 5 School of Medicine, University College Dublin, Dublin, Ireland; 6 Department of Intensive Care, King’s College London, Guy’s and St Thomas’ Hospital, London, UK; 7 Department of Medicine, University of Florida, Gainesville, FL USA; 8 Intelligent Critical Care Center, University of Florida, Gainesville, FL USA; 9 Department of Medicine, University of California, San Diego, CA USA; 10 Center for Acute Care Nephrology, Cincinnati Children’s Hospital Medical Center, Cincinnati, OH USA; 11 Division of Nephrology and Hypertension, Division of Pulmonary and Critical Care Medicine, Department of Medicine, Mayo Clinic, Rochester, MN USA; 12 Department of Pharmacy and Therapeutics, School of Pharmacy, University of Pittsburgh, Pittsburgh, PA USA; 13 Department of Pharmacy, UPMC, Pittsburgh, PA USA; 14 Department of Critical Care Medicine, Department of Biomedical Informatics, School of Medicine and the Clinical Translational Science Institute, University of Pittsburgh, Pittsburgh, PA USA

**Keywords:** Acute kidney injury, Drug-related side effects and adverse reactions, Adverse drug event, Critically ill, Critical care, Intensive care units

## Abstract

Drug-induced kidney disease (DIKD) accounts for about one-fourth of all cases of acute kidney injury (AKI) in hospitalized patients, especially in critically ill setting. There is no standard definition or classification system of DIKD. To address this, a phenotype definition of DIKD using expert consensus was introduced in 2015. Recently, a novel framework for DIKD classification was proposed that incorporated functional change and tissue damage biomarkers. Medications were stratified into four categories, including “dysfunction without damage,” “damage without dysfunction,” “both dysfunction and damage,” and “neither dysfunction nor damage” using this novel framework along with predominant mechanism(s) of nephrotoxicity for drugs and drug classes. Here, we briefly describe mechanisms and provide examples of drugs/drug classes related to the categories in the proposed framework. In addition, the possible movement of a patient’s kidney disease between certain categories in specific conditions is considered. Finally, opportunities and barriers to adoption of this framework for DIKD classification in real clinical practice are discussed. This new classification system allows congruencies for DIKD with the proposed categorization of AKI, offering clarity as well as consistency for clinicians and researchers.

## Introduction

Acute kidney injury (AKI) occurs in about 10–15% of hospitalized and more than 50% of intensive care unit (ICU) patients [1]. Severity of acute and chronic illnesses, iatrogenic exposures, and discrepant AKI definitions lead to variability in prevalence estimates across populations and across studies. Still, mortality rates reach up to 65% in the ICU for patients with AKI [2].

Clinicians and researchers have classified the heterogeneous etiologies of AKI using “pre-renal,” “renal/intra-renal,” and “post-renal” categories to explain the nature of the kidney insult [3]. However, this approach fails to account for the overlapping and dynamic nature of AKI due to various etiologies including drugs. For example, a patient with AKI in the context of a decreased effective arterial blood volume from over diuresis—referred to as “pre-renal” AKI, can progress to parenchymal damage if this scenario is prolonged for an extended duration or if another drug such as a nonsteroidal anti-inflammatory drug (NSAID) administered in combination during this pre-rental state, subsequently referred to as “intra-renal.” Leading consensus groups recommend a more explicit and comprehensive AKI classification based on evidence of kidney dysfunction and/or damage. The 10th and 23rd Acute Disease Quality Initiative (ADQI) working groups proposed the terms “functional AKI” and “kidney damage” instead of “pre-renal,” “renal,” and “post-renal”. This working group also suggested to exploit both functional and damage kidney biomarkers along with non-kidney biomarkers (e.g., natriuretic peptides, procalcitonin) to better define AKI and characterize its etiologies [4]. Furthermore, the 23rd ADQI expert panel suggested to subcategorize KDIGO stage 1 AKI into 3 substages (1S, 1A, and 1B) and stage 2 and 3 individually into 2 substages (2A & 2B and 3A & 3B, respectively) based on the measurement of functional and damage biomarkers [5].

There is not a standard definition or classification system for drug-induced kidney disease (DIKD). In addition, the application of novel AKI stages and substages to classify DIKD has not been described previously. Clarity in classification is needed for DIKD because consistency with contemporary categorization allows for effective communication across various AKI etiologies and this is most important for nephrotoxic drugs due to their frequent association with AKI in critically ill patients [2]. Therefore, in this perspective article, we: (1) provide an innovative framework for DIKD classification based on previous work from the 23rd ADQI conference; (2) outline the role of novel kidney biomarkers in this staging system; and (3) suggest possible opportunities as well as potential pitfalls for its adoption into clinical practice, especially in critically ill patients.

## Drug-induced kidney disease

**Fig. 1:**
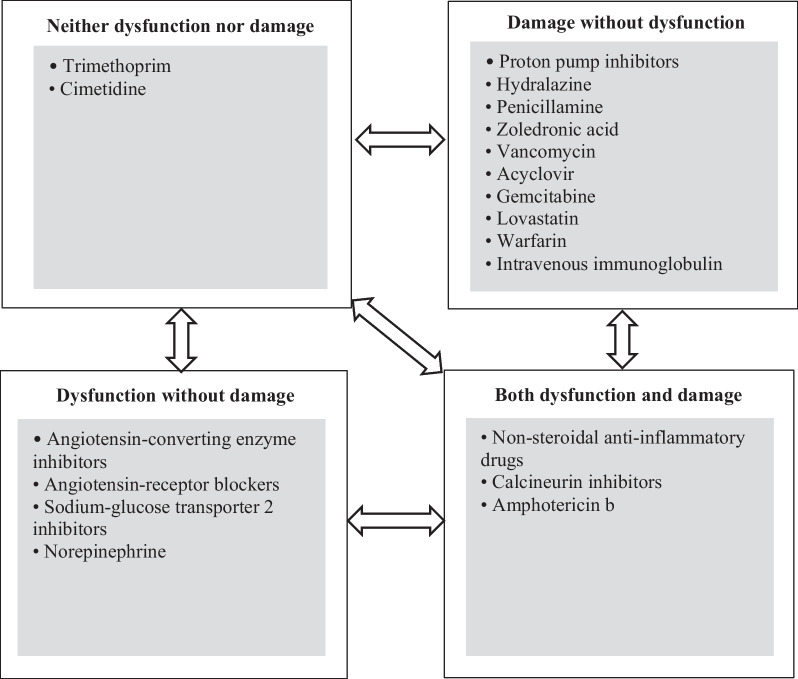
Classification system of drug-induced kidney injury based on functional and damage biomarkers suggested by Ostermann et al. [10]. Since most experts prefer the term “damage” to “injury” for describing the pathology and pathophysiology of AKI induced by different etiologies such as medications, we have replaced “injury” with “damage” in the title of each DIKD category introduced primarily by the ADQI expert group [10]. Drug or drug class examples for each category have been provided just to clarify more this classification system of DIKD. Listed medications are only examples of each category, and they should not be considered all-inclusive. In the presence of susceptibility factors, medications belonging to “neither dysfunction nor damage group” can move to other categories. This is also true for the “dysfunction without damage” and “damage without dysfunction” categories. Arrows depict the possible movements between categories. The movement of a given patient at a specific time course from the “damage without dysfunction” category to the “dysfunction without damage” category or vice versa makes no sense from pharmacological and clinical perspectives. The bidirectional arrows mean that both the progression and recovery of DIKD are possible in certain categories

One-fourth of all medications given in hospitals are potentially nephrotoxic [6]. DIKD is estimated to account for 19–26% of all cases of AKI in hospitalized patients [7], with medications among the most common causes of AKI in ICU patients [8]. Studies have variably used the classification systems mentioned previously, as well as attempted to integrate temporality and mechanism of injury into DIKD assessment [9]. In 2015, Mehta et al. suggested four phenotypes of DIKD (AKI, glomerular disorder, nephrolithiasis, and tubular dysfunction) based on clinical presentation/mechanism of injury. Considering conceptual models about time course of events, DIKD was further classified into acute (1–7 days), sub-acute (8–90 days), and chronic (> 90 days) [7]. Recently, a novel framework (2 × 2 table) classification was proposed by an expert panel at the 23rd ADQI conference held in April 2019 in Rome, Italy (Fig. 1) [10]. Authors of this perspective article, some of whom were participants of the 23rd ADQI, contextualize the framework for DIKD based on their opinion to provide clarity and application for DIKD [10]. Accordingly, both functional and damage biomarkers along with predominant mechanism(s) of nephrotoxicity have been integrated to classify medications into the following four categories: dysfunction without damage, damage without dysfunction, dysfunction and damage together, and neither dysfunction nor damage (Fig. 1) [10].

### Neither dysfunction nor damage

#### Mechanisms

Medications that cause an increase in serum creatinine (SCr) without causing renal dysfunction or damage based on the current knowledge inevitably fit into this category. For instance, certain medications can decrease tubular secretion of creatinine, interfere with the creatinine assay, or enhance creatinine production without changing kidney function. To the current knowledge, these drugs are not nephrotoxic, and this clinical situation is referred to as pseudo-AKI. Competition with creatinine secretion at the proximal tubule, mediated via drug efflux transporters such as organic cation transporter, is a major proposed mechanism for medications of this category. These changes in SCr are independent of any significant alteration in GFR or damage to the glomeruli, tubules, or interstitium of the kidney. Accordingly, this category can also be called “SCr elevations without dysfunction or damage.”

#### Drugs/drug classes examples

Medications causing a rise in SCr in the absence of kidney dysfunction or damage (i.e., pseudo-AKI) belong to this category (Table 1) [11–16]. They are related to different drug classes, primarily antibiotics and antineoplastics. Cimetidine, cobicistat, dolutegravir, trimethoprim, olaparib, and imatinib are examples of drugs interfering with tubular secretion of creatinine.

**Table 1:** List of drugs/drug classes associated with pseudo-AKI and their possible mechanism(s)

Drugs/drug classes	Mechanism(s)
*Antibiotics/antivirals:*^†^ Trimethoprim^‡^, pyrimethamine, cobicistat, dolutegravir *Antiarrhythmics:* Dronedarone *Gastrointestinal agents:* Cimetidine *Antineoplastics:* Tyrosine kinase inhibitors (e.g., imatinib, bosutinib, sorafenib, sunitinib, crizotinib, gefitinib, and pazopanib) Poly-ADP-ribose polymerase inhibitors (e.g., olaparib, niraparib talazoparib) Cyclin-dependent kinase 4/6 inhibitors (e.g., palbociclib, abemaciclib, ribociclib) Anaplastic lymphoma kinase inhibitors (e.g., crizotinib, ceritinib, alectinib, brigatinib, lorlatinib) *Others:* Probenecid	Competing with and decreasing proximal tubule creatinine secretion in a dose-dependent manner, mediated via the inhibition of drug efflux transporters such as organic cation transporter
Creatine supplements (both short term and long term)	Increasing the precursor of creatinine
Corticosteroids	Increasing catabolic state is associated with the release of creatine from muscle, spontaneously converted to creatinine Some formulations of dexamethasone may contain creatinine as a buffer excipient.
Azasetron	Some formulations may contain creatinine as a buffer excipient.
Fenofibrate	Increasing the metabolic production of creatinine
Cephalosporins (e.g., cephalothin, cefazolin, cephalexin, cefoxitin, cefaclor, cephradine), clavulanic acid	Interfering with the analytical measurement of creatinine (Jaffe method)
5-Flucytosine	Interfering with the analytical measurement of creatinine (Ektachem enzymatic system)
Calcitriol and alfacalcidol	Unknown

^†^Pseudo-AKI via inhibiting organic anion transporters 1 & 3 in proximal tubule has also been reported with antibiotic combinations such as piperacillin/tazobactam or vancomycin plus piperacillin/tazobactam [17, 18]. Nevertheless, interstitial nephritis due to piperacillin/tazobactam has been documented in the literature [19, 20]. Thus, it seems prudent not to place piperacillin/tazobactam into the “neither dysfunction nor damage” category

^‡^Sulfamethoxazole/trimethoprim should be discriminated from trimethoprim alone and viewed as an independent agent because of described interstitial nephritis or crystal nephropathy cases induced by sulfamethoxazole/trimethoprim [21]. Therefore, sulfamethoxazole/trimethoprim may not match the “neither dysfunction nor damage” category

#### Diagnostic biomarkers

In the case of pseudo-AKI caused by medications such as cobicistat, SCr concentrations can increase by about 0.2–0.4 mg/dL [20]. Similarly, dolutegravir recipients may experience a modest, non-progressive SCr increase (about 0.14 mg/dl) within two weeks after initiation of therapy [22]. This increase in SCr does not correspond with a decrease in true GFR, measured using iohexol [23, 24]. Novel kidney function biomarkers, such as serum cystatin C, can assist in identifying pseudo-AKI caused by medications and differentiate it from true AKI [16]. In contrast to SCr, serum cystatin C level is expected to be normal in the setting of pseudo-AKI. In this regard, a recently published prospective cohort study in 739 critically ill patients demonstrated that vancomycin plus piperacillin/tazobactam recipients had significantly higher creatinine-defined AKI rates than those who received vancomycin plus cefepime combination. In contrast, alternative biomarkers such as serum cystatin C and blood urea nitrogen were comparable between the two groups [25]. Interestingly, clinical outcomes including dialysis or mortality did not differ significantly between vancomycin plus piperacillin/tazobactam and vancomycin plus cefepime combination recipients. However, since glomerular filtration rate (GFR) was not directly measured, it is unclear whether SCr was overly sensitive (or falsely elevated) or if serum cystatin C was insensitive to AKI from this drug. Furthermore, when AKI occurred in patients receiving piperacillin/tazobactam, vancomycin, or both, urinary tissue inhibitor of metalloproteinases-2 (TIMP-2) and Insulin-like growth factor binding protein 7 (IGFBP7) increased, suggesting that the observed increase in SCr after administration may represent injury [26].

### Dysfunction without damage

#### Mechanisms

A group of medications may lead to deterioration in kidney function without direct glomerular or tubular damage, such medications may act on systemic or intraglomerular hemodynamics [27]. In the case of no or inadequate compensation, altered renal blood flow and kidney perfusion can decrease intraglomerular pressure, decrease filtration fraction and GFR, and increase SCr concentration corresponding with a decrease in estimated GFR (eGFR) [10, 28]. In some cases, this change in glomerular hemodynamics may be an adverse event; in others, it may be the therapeutic intent.

#### Drugs/drug classes examples

Decreased effective arterial blood volume from systemic vasodilatory use or over diuresis with furosemide or mannitol resulting in hypovolemia may elicit a decrease in kidney perfusion and the resultant increase in SCr [29, 30]. Treatment with vasoactive agents such as norepinephrine and vasopressin may lead to excessive systemic vasoconstriction, which may similarly decrease kidney perfusion and result in an increase in SCr [30]. This condition should be considered separately from certain clinical contexts (e.g., fluid-resuscitated, hyperdynamic sepsis) when vasoactive drugs can preserve renal blood flow and GFR. Reasons why diuresis or excessive systematic vasoconstriction occur include the interpatient variability in response to treatment, the dynamic nature of the patient’s condition, or unintentional medication errors. Achieving the desired fluid balance and optimizing hemodynamics in a critically ill patients require a fine balance with continuous monitoring to achieve a targeted, personalized response.

Angiotensin converting enzyme inhibitors (ACEIs) and angiotensin II receptor blockers (ARBs) preferentially dilate the efferent arteriole, leading to reduced intraglomerular pressure, rather than an overall decrease in renal blood flow [21]. In the absence of severe bilateral renal artery stenosis (unilateral in solitary kidney patients) or volume depletion, exposure to ACEIs/ARBs can lead to an increase in SCr up to 30% from baseline values (with a corresponding decline in eGFR) within the first and second week of treatment. These are generally considered acceptable hemodynamic changes and usually reversible upon stopping ACEIs/ARBs [21]. A similar pattern of increasing SCr is observed during the early phase of treatment with sodium–glucose cotransporter 2 inhibitors (SGLT2is). For ACEIs/ARBs and SGLT2is, this phenomenon has been labeled as either “permissive AKI” or “permissive hypercreatinemia” [31, 32]. In other words, long-term nephron/cardio protection with ACEIs/ARBs and SGLT2is (e.g., reducing proteinuria and delaying chronic kidney disease [CKD] progression) mostly outweighs mild decreases in GFR during the early phase of treatment.

#### Diagnostic biomarkers

**Fig. 2:**
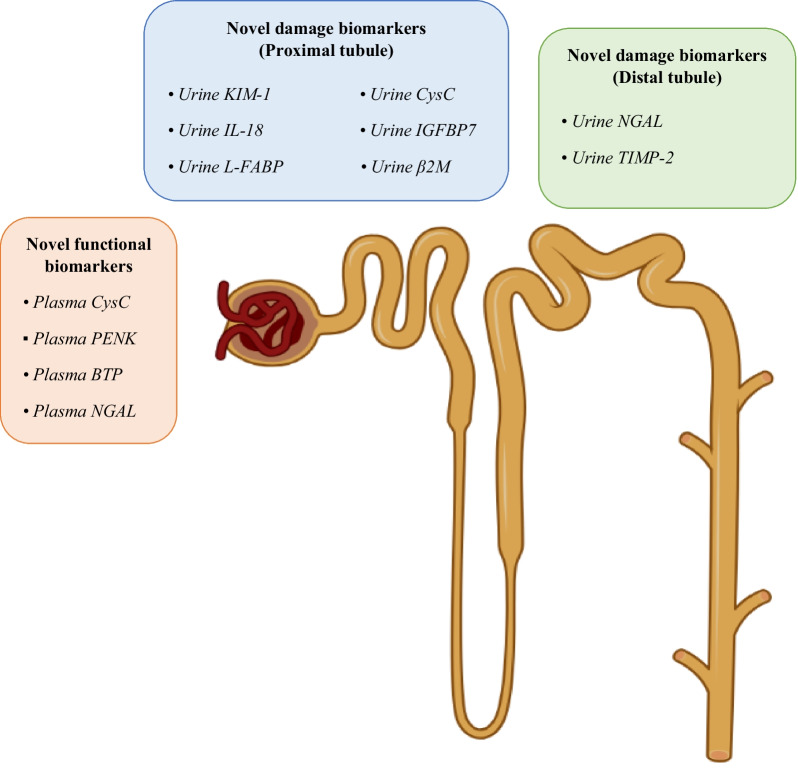
Some novel functional and damage biomarkers of the kidney can help to classify drug-induced kidney disease based on the suggested framework by Ostermann et al. [10]. Damage biomarkers are related to different sites of the kidney and are mostly site-specific. KIM-1 is a cell membrane glycoprotein upregulated in the presence of nephrotoxic/ischemic damage to proximal tubule epithelial cells. NGAL is a glycoprotein expressed in various tissues, including the kidney. Its expression is markedly upregulated after kidney ischemia. IL-18 is a pro-inflammatory cytokine expressed during proximal tubular injury. L-FABP is expressed in the proximal tubule, and its expression is augmented by hypoxic stress. β2M is a low molecular weight polypeptide that presents on the cell surface of all nucleated cells. In the case of tubular dysfunction, its level in urine will increase. TIMP-2 and IGFBP7 are preferentially expressed and secreted from distal and proximal tubules, respectively, in response to stress and damage. All these damage biomarkers have preliminary clinical evidence that is promising. Urinary KIM-1 and NGAL have the most clinical evidence in the setting of drug-induced kidney disease  [42]. Besides these two biomarkers, urinary IL-18, L-FABP, and TIMP-2·IGFBP7 can also help diagnose ATI early and differentiate injury from dysfunction. Urinary TIMP-2·IGFBP7 appears to be an appropriate candidate damage biomarker of DIKD, particularly in preoperative and critically ill settings, because of its features discussed elsewhere [43, 44]. Three novel functional biomarkers in serum have been introduced and studied in clinical settings. CysC is a low molecular weight protein produced by all nucleated cells and cleared only by glomerular filtration [41]. PENK is the precursor polypeptide hormone of the enkephalin family freely filtered in the glomerulus [40]. BTP is a small protein primarily produced in the cerebral fluid and eliminated by glomerular filtration [41]. Except for plasma CysC, there are currently no clinical data on other novel functional biomarkers of the kidney associated with medications. Despite promising findings with novel functional and damage biomarkers, they should be interpreted cautiously because AKI, the primary endpoint in these studies, is mostly diagnosed by changes in serum creatinine concentration rather than specific biomarkers. *CysC,* Cystatin C; *PENK*, Proenkephalin A; *BTP*, β-trace protein; *KIM-1*, Kidney injury molecule-1; *NGAL*, Neutrophil gelatinase-associated lipocalin; *IL-18*, Interleukin-18; *L-FABP*, Liver-type fattyacid-binding protein; *β2M,* Beta-2 microglobulin; *IGFBP7,* Insulin-like growth factor binding protein 7; *TIMP-2,* Tissue inhibitor of metalloproteinases-2

Laboratory data are required to identify and quantify kidney dysfunction in the setting of DIKD. SCr and urine output are the classic biomarkers to characterize kidney dysfunction [33]. Owing to the limitations of these standard tools [34], novel functional markers have been identified [35], for example, serum cystatin C. Serum cystatin C is devoid of many limitations of SCr, i.e., it is not affected by muscle mass, diet, sex, or tubular secretion. Serum cystatin C has been proposed to be more sensitive and specific than SCr for detecting GFR changes. For example, a meta-analysis of 30 prospective cohort trials comprising 4,247 adult patients from 15 countries, of which 28.5% were in ICU/cardiac care units revealed that the overall diagnostic sensitivity and specificity of serum cystatin C was 0.82 (95% CI 0.75 to 0.87) and 0.82 (95% CI 0.78 to 0.86), respectively. The area under the receiver operating characteristic curve (AUROC) for diagnostic accuracy of serum cystatin C for AKI was 0.89 [36]. In addition, the combination of SCr and cystatin C-based formula significantly improved target trough achievement of vancomycin compared to estimated creatinine clearance among ICU patients with stable kidney function [37]. Even so, prediction equations appear not to have the same validation for GFR changes in AKI compared to CKD [38, 39]. Plasma proenkephalin A (PENK) is another functional kidney biomarker of interest with better accuracy to estimate GFR and detect AKI compared to SCr, particularly in critically ill patients with sepsis or septic shock [40]. Integrating β-trace protein and β2-microglobulin into GFR estimation equations as a panel of functional markers provides higher accuracy for various kidney disorders [41] (Fig. 2).

### Damage without dysfunction

#### Mechanisms

Kidney damage within this class may be glomerular, tubular, or interstitial (Table 2). For example, in terms of drug-induced tubular damage/injury directly and indirectly (e.g., crystals and casts), oxidative stress and inflammation play pivotal roles [45]. Mitochondrial dysfunction is another common mechanism of drug-induced tubular damage/injury, leading to adenosine triphosphate (ATP) depletion and finally cell death [46]. Immune reactions, mostly mediated by T-cells and complement activation, account for major features of acute tubulointerstitial nephritis secondary to medications [45, 47]. This damage can be either intrinsic (predictable and dose-dependent) or idiosyncratic (unpredictable and dose-independent). For this framework, drug classification is based on the predominant mechanism of nephrotoxicity, and for “damage without dysfunction” if not detected and managed early, transition to “dysfunction and damage together” may occur. Concurrent kidney dysfunction, as outlined in “Dysfunction without damage” section, could be either absent or undetectable by present clinical/laboratory methods, particularly in the initial phases of kidney damage or when patients have normal baseline kidney function and thus plentiful functional renal reserve. Kidney dysfunction may develop and become evident later during the use of this category of medications (refer to “Dysfunction and damage together” section).

**Table 2:** Glomerular, tubular, and interstitial damage to the kidney caused by medications

Type of damage	Examples
*Glomerular/vascular*	
Minimal change disease	Interferon alpha & beta, Lithium
Focal segmental glomerulosclerosis	Bisphosphonates (especially pamidronate and zoledronic acid), Lithium
Membranous nephropathy	Penicillamine, Anti-tumor necrosis factor agents
Vasculitis	Hydralazine, Propylthiouracil, Allopurinol, Phenytoin, Penicillamine, Minocycline
Thrombotic microangiopathy	Gemcitabine, Bevacizumab, Interferon alpha, Ticlopidine, Clopidogrel, Oral contraceptives
Cholesterol emboli	Warfarin, Streptokinase, Alteplase
*Tubular*	
Acute tubular injury/necrosis	Aminoglycosides, Vancomycin, Colistin, Foscarnet, Pentamidine, Tenofovir, Cisplatin, Carboplatin, Zoledronic acid
Fanconi syndrome	Tenofovir, Sodium valproate, Deferasirox
Obstructive nephropathy	Acyclovir, Sulfonamides, Methotrexate, Indinavir, Atazanavir, Triamterene, Sodium phosphate
Rhabdomyolysis	Lovastatin, Simvastatin
Tumor lysis syndrome	Cytotoxic agents, Glucocorticoids
Osmotic nephrosis	Intravenous immunoglobulin, Hydroxyethyl starch
Nephrogenic diabetes insipidus	Lithium
Nephrogenic syndrome of inappropriate antidiuresis	Carbamazepine, Haloperidol, Cyclophosphamide, Selective serotonin reuptake inhibitors
*Interstitial*	
Acute tubulointerstitial nephritis	Beta-lactam antibiotics, Rifampin, Aminosalicylates, Proton pump inhibitors
Chronic tubulointerstitial nephritis	Lithium, Aristolochic Acid

The prevailing etiology of nephrotoxicity of these medications or medication classes is direct/indirect toxic effects on the kidney. Therefore, they mostly belong to the “damage without dysfunction” category. Notably, these medications may have more than one aspect of kidney damage. For example, apart from glomerular changes, cholesterol emboli induced by warfarin, streptokinase, or alteplase can also affect the peritubular microcirculation, leading to tubular damage. Similarly, in the case of crystal nephropathy caused by medications, both direct and indirect mechanisms are usually involved. Therefore, this table lists only some examples of medications/medication classes and should not be considered conclusive. In addition, this table does not consider medications with both damage and dysfunction features such as nonsteroidal anti-inflammatory drugs, amphotericin B, and calcineurin inhibitors

#### Drugs/drug classes examples

The most reported mechanism and presentation of DIKD in the inpatient setting is usually acute tubular injury (ATI) [21]. Nevertheless, acute/chronic interstitial nephritis alone or in association with other aspects of kidney damage is also quite frequent among cases of DIKD, especially in pathology reports; however, it may be missed and under-diagnosed in clinical practice, mostly due to lack of overt signs and symptoms [33, 48]. For example, the prevailing mechanism of nephrotoxicity related to commonly used antibiotics in hospitals such as aminoglycosides and vancomycin is direct tubular damage, mostly ATI. Additional relevant examples are provided in Table 2 [21, 49, 50].

#### Diagnostic biomarkers

The prevailing feature of the “damage without dysfunction category” of DIKD is the presence or increase of damage biomarkers in plasma or urine, with preserved kidney function. The release of kidney damage biomarkers is typically more rapid than elevation of SCr which may take 36–72-h after the onset of kidney damage. The rise in damage biomarkers without increased SCr has been described as “subclinical AKI.” This phenomenon is particularly prominent when baseline kidney function is normal [51]. Finally, SCr is a crude biomarker of kidney function/injury as it does not localize the site of DIKD within the kidney nor reveal underlying causes. Besides SCr, proteinuria, and albuminuria, often quantified by urine albumin to creatinine ratio, are considered other conventional biomarkers of kidney damage. Clinical studies have demonstrated their performance in detecting drug-induced glomerular and/or tubular injury (e.g., cisplatin) [41, 51]. Nevertheless, these biomarkers are prone to intraindividual variability because of non-kidney factors including protein intake and exercise.

In 2018, the US Food and Drug Administration (FDA) qualified six novel urine biomarkers in conjunction with traditional measures of kidney function for use in medical product development and regulatory review to aid in the detection of kidney injury in phase 1 trials where there is concern for a drug causing kidney injury [52]. These include urinary clusterin, kidney injury molecule-1 (KIM-1), *N*-acetyl-beta-d-glucosaminidase (NAG), neutrophil gelatinase-associated lipocalin (NGAL), osteopontin, and cystatin C. In addition, interleukin-18 (IL-18), liver-type fatty-acid-binding protein (L-FABP), TIMP-2, and IGFBP7 in urine are considered markers of kidney damage [26, 42]. They can detect patients with or at risk for DIKD [50]. The product of the two markers TIMP-2 and IGFBP7 has FDA-approval as the Nephrocheck® test [42].

### Dysfunction and damage together

#### Mechanisms

This category encompasses medications associated with hemodynamically- and non-hemodynamically related AKI mechanisms [10]. Non-hemodynamic features of AKI include glomerular, tubular, and interstitial injury alone or in combination. Notably, a drug may produce changes in function through one mechanism and tissue damage through another, or both may arise through the same mechanism. Furthermore, the severity of dysfunction and damage may not be equivalent, possibly causing a patient to move from one category to another, such as “dysfunction without damage” followed by “damage without dysfunction.” In other words, different mechanisms and aspects of AKI as a result of these medications may not coincide, and therefore, the time sequence of events should be considered.

#### Drugs/drug classes examples

Prototypes of this category are non-steroidal anti-inflammatory drugs (NSAIDs) which may contribute to DIKD through (1) decreasing overall renal blood flow and intraglomerular pressure secondary to afferent arteriole vasoconstriction, (2) ATI, (3) acute tubulointerstitial nephritis, (4) glomerular injury (e.g., minimal change disease or membranous glomerulonephritis), and (5) papillary necrosis [7, 21]. Other example in this category is calcineurin inhibitors. Calcineurin inhibitor nephrotoxicity is associated with afferent arteriole vasoconstriction, leading to overall reduction of renal blood flow and intraglomerular pressure (hemodynamic component), thrombotic microangiopathy as well as focal segmental glomerulosclerosis (glomerular and tubular components), and chronic tubulointerstitial nephritis or fibrosis (interstitial component) [21, 53]. Similarly, amphotericin B deoxycholate contributes to mixed injury and dysfunction [21, 54]. There is not an explicit predominant mechanism of injury to assign these drugs to one of the other categories.

#### Diagnostic biomarkers

For this type of DIKD, functional and damage biomarkers are pivotal in prediction, diagnosis, and prognostication. Apart from SCr as a classic biomarker of kidney function and damage, novel functional biomarkers of potential value to identify DIKD from this category of medications include serum cystatin C, proenkephalin A, and β-trace protein. In addition, serum and, specifically, urinary kidney stress or damage markers can assist in differentiating this category from DIKD due to “dysfunction without damage” (Fig. 2).

## Movement between categories

This contemporary classification system broadly focuses on placing a drug in a category using the predominant mechanism of injury or dysfunction in the individual. Still, patient-specific scenarios may present atypically, and there may be a shift from one category to another as a DIKD event progresses or resolves. Notably, this new 2 × 2 classification system of DIKD allows for movement between categories (Fig. 1) that is not considered in the traditional pre/intra/post-renal classification system. Susceptibilities and exposures are context-specific such as volume depletion, advanced age, underlying kidney disease, and diabetes mellitus may catalyze or accelerate the transformation between categories [10]. For example, treatment with ACEIs or ARBs that reduce intraglomerular pressure, when coupled with volume depletion from a concurrent diuretic (e.g., furosemide) especially in elderly patients with heart failure, may increase SCr by more than 30% from baseline values. In this scenario, tubular injury may occur secondary to reduced oxygen delivery to the kidney parenchyma [55].

Similarly, in addition to altered glomerular hemodynamics caused by SGLT2is, co-administering NSAIDs or cyclosporine with these agents or the presence of volume depletion secondary to excessive fluid loss (e.g., nausea, vomiting, diarrhea) can also lead to kidney medullary hypoxia and injury, finally evolving into ATI [56]. In each of these cases, DIKD would progress from “dysfunction without damage” to the “dysfunction and damage together” category (Fig. 1). Importantly, this condition should be viewed and interpreted as bidirectional, depending on the comorbidities of the individual patient. Regarding aminoglycosides, their use in patients with obstructive jaundice or co-treatment with NSAIDs may alter renal hemodynamics by decreasing kidney blood flow. This can potentially reduce drug elimination and increase the intra-tubular concentration of aminoglycosides, eventually leading to enhanced aminoglycoside nephrotoxicity [57]. Thus, DIKD may transition from the “damage without dysfunction” category to “dysfunction and damage together.”

The pathogenesis and severity of DIKD are commonly multifactorial, combining predisposing risk factors with exposure to nephrotoxin(s) and other insults. For example, in the case of medications belonging to the “both injury and dysfunction” category, the complete picture and different aspects of nephrotoxicity usually may not be observed unless other medication and non-medication related risk factors are present. For NSAID nephropathy, some of these factors are age above 60 years, true volume depletion (secondary to dehydration), effective arterial volume depletion (secondary to congestive heart failure, cirrhosis, and nephrotic syndrome), and concurrent treatment with ACEIs/ARBs, diuretics, or calcineurin inhibitors [31, 58]. In critically ill patients, the simultaneous presence of these factors is highly probable. Notably, in the case of concurrent treatment with ACEIs/ARBs, these agents can worsen NSAID-mediated reductions in oxygen delivery to the kidney parenchyma. Medication-induced crystalline nephropathy is another example. It varies from simple urine crystallization without kidney involvement (neither dysfunction nor damage) to full-blown kidney involvement (dysfunction and damage together), depending on the possible presence of volume depletion, drug dosing, urine pH, and underlying kidney disease [59]. Therefore, it is likely that patients who received a specific nephrotoxic medication may be considered for different DIKD categories, depending on risk factors.

The potential of transition between categories provides opportunities for clinical management. A preliminary report in kidney transplant recipients demonstrated that in patients who developed cyclosporine nephrotoxicity, increased urine β2-microglobulin concentrations were detectable proceeding a SCr rise. Interestingly, cyclosporine dose reduction in these patients led to decreased urine β2-microglobulin [60]. In addition, based on results of a cohort investigation, urinary NGAL levels between 96 and 144 h and urinary [TIMP-2]·[IGFBP7] normalized by urinary creatinine between 144 and 192 h of vancomycin use were predictors of developing AKI during hospital stay and recovery of AKI at the time of hospital discharge, respectively [61]. There is the possibility of tracking patients’ improvement with DIKD using novel biomarkers. A better understanding of DIKD severity will require researchers to evaluate daily biomarker concentrations, so that trends can be monitored closely; however, most current studies evaluate biomarker concentrations only intermittently.

Movement between categories may be bidirectional. Therefore, apart from progression, this model also predicts the possible partial or complete recovery of DIKD. Accordingly, the impact of co-administering agents with potential nephroprotective properties on DIKD can be described. For instance, concurrent oral n-acetyl cysteine therapy (600 mg twice a day) significantly reduced the rate of amphotericin B nephrotoxicity as defined by alteration in SCr and eGFR in patients with different infectious diseases [62]. This observation can be interpreted as the movement of patients from the “dysfunction and damage together” category to one of the other three categories.

## Opportunities, barriers, and clinical adoption

The traditional classification of DIKD, which relied on anatomical considerations, had several limitations. The proposed refined pathophysiological staging system, like suggestions by Mehta et al. [8], may address some of the current questions and complexities of DIKD. Advantages of the proposed 2 × 2 framework include dynamic movement between categories independent and devoid of anatomical constraints, and use of both kidney (classical and novel) and non-kidney biomarkers (e.g., natriuretic peptides such as B-type natriuretic peptide [BNP] or N-terminal pro B-type natriuretic peptide [NT-proBNP], C-reactive protein [CRP]). The aggregate use of clinical features and broad application of biomarkers provides an opportunity for early detection and management of DIKD, determination of the pathophysiological mechanism(s) of DIKD, and an understanding of the possible relationships between different phases of DIKD and other causes of AKI.

Importantly, the proposed 2 × 2 framework for DIKD has limitations. Susceptibility factors that catalyze progression have been suggested, but protective factors to facilitate recovery are unclear. In addition, although this framework has been depicted as four distinct categories, it seems prudent to consider and interpret the model as a continuum from subclinical to clinical AKI [4]. Classifying drugs or drug classes into one single category is challenging, but focusing on the primary mechanism of kidney injury guides this process. For example, ACEI/ARBs predominately contribute to hemodynamic changes within the kidney (i.e., “dysfunction without damage” category), but high-dose captopril has also been associated with membranous nephropathy [63]. SGLT2is would customarily be allocated to the “dysfunction without damage” category, but direct tubular injury caused by uricosuria or glycosuria may be possible with these agents, too [21]. Another example is drug-induced crystalline nephropathy by direct (obstructive) and indirect (non-obstructive) mechanisms. Accordingly, tubular damage caused by drug crystals could lead to intra-tubular obstruction in the early phase. On the other hand, some medications and/or their metabolites can increase intra-tubular pressure. This leads to decreased filtration pressure, kidney blood flow and GFR, and increased SCr concentration after 24–48 h [64]. If these detrimental processes persist and are not corrected promptly, they may eventually result in both tubular and interstitial injury due to inflammation and necroinflammation within the kidney [13, 59, 64]. Even in the case of aminoglycoside nephrotoxicity where ATI is the prominent presentation (i.e., “damage without dysfunction”), concurrent or subsequent vascular effects can decrease renal blood flow and consequently, reduce GFR (i.e., “damage and dysfunction together”); this can appear sequentially or concurrently in presentation [65]. Considering the limits of categorizing a drug in this framework is critical, but not moving forward with contemporary approaches would have greater restrictions.

Importantly, it seems reasonable to consider only the predominant mechanism(s) of nephrotoxicity for classifying medications to limit unwanted variance when using this framework in clinical practice. Although medications/medication classes provided in “Damage without dysfunction” section and Table 2 have prominent direct/indirect toxic effects on the kidney via different mechanisms, their possible role in causing concurrent kidney dysfunction cannot be easily ruled out or differentiated. Prevailing mechanism(s) should be based on high-quality evidence. Determining the predominant mechanism of DIKD in clinical practice is challenging and not commonly determined, so dependence on published evidence for probabilistic assessment is a realistic strategy. In addition, new information on the pathophysiology of DIKD should be considered, and possible exceptions in each category should also be kept in mind.

Finally, the framework does not provide direction about the possibility and severity of nephrotoxicity of different medications. The framework needs to relate to and be used along with nephrotoxicity rating systems such as the appraised nephrotoxic potential (NxP), to allow for better categorization and prioritization of nephrotoxin stewardship, especially in the ICU. Interestingly, the clinical utility of NxP has been recently demonstrated in determining the potential nephrotoxicity of 167 drugs used in adult critically ill patients. Twenty drugs such as analgesics (NSAIDs) and anti-infectives (e.g., amikacin, tobramycin, colistin, foscarnet, vancomycin) were considered to have probable to definite nephrotoxicity [66].

The performance of novel biomarkers in identifying different aspects and determining their possible time sequence of AKI during DIKD, especially in the case of medications belonging to “both dysfunction and damage category” such as NSAIDs or amphotericin B, should be addressed in future studies. Discriminating medications from other possible causes of AKI, such as sepsis, may challenge the specificity of these biomarkers in detecting DIKD in real clinical practice [67]. Therefore, apart from novel functional and damage kidney biomarkers and non-kidney biomarkers briefly mentioned above, other laboratory findings such as urine microscopy examination, imaging, metabolic and proteomic analysis, and exosomal assessments may have an important role to play in differentiating different DIKD categories. Clinical studies need to assess possible movement between different categories of DIKD by using novel kidney biomarkers. Importantly, the use of biomarkers to assist in determining AKI etiology requires easy access and quick results for clinical application.

Regarding the commercial availability of novel biomarker assays, NGAL and L-FABP testing kits are generally available for both research and clinical/diagnostic uses in the USA and Europe. On the other hand, KIM-1 and IL-18 availability in the USA are officially limited to research uses. [TIMP-2] and [IGFBP7] are available for clinical/diagnostic uses in the USA and Europe [42].

## Summary

We offer an innovative, contemporary framework for DIKD classification that uses a conceptual 2 × 2 table to integrate functional and damage markers in the assessment of DIKD. This classification system allows DIKD to be consistent with the proposed categorization of AKI, offering clarity and consistency for clinicians and researchers, especially in critically ill patients, where multiple comorbidities and possible confounders exist. Novel biomarkers also drive the need to change the way with think about our traditional AKI and DIKD classification as we may more readily determine kidney damage earlier than dysfunction allowing for timely intervention. The contemporary framework may also be a tool to aid clinicians in explaining to patients and caregivers why some drugs should be continued despite the development of a decrease in GFR and why other medications can be restarted even if AKI is still present. Still, while a given drug may be classified according to its most typical characteristics, a specific patient’s case of DIKD may be a less common manifestation. Therefore, clinicians need to have a comprehensive view of DIKD classification and try to treat the patient, not the drug.

## Data Availability

Not applicable.
